# Anti-ganglioside antibody positive neuromyelitis optica spectrum disorders with peripheral neuropathy: a case report

**DOI:** 10.1186/s12883-023-03214-6

**Published:** 2023-05-12

**Authors:** Yangchun Li, Man Tang, Lu Yu, Ying He, Lisong Liang, Hao Qu, Wei Si, Xiao Hu

**Affiliations:** 1 Department of Neurology, Guizhou Provincial People’s Hospital, Guiyang, 550002 China; 2 Department of Electrophysiology, Guizhou Provincial People’s Hospital, Guiyang, 550002 China; 3 Department of Radiology, Guizhou Provincial People’s Hospital, Guiyang, 550002 China

**Keywords:** Neuromyelitis optica spectrum disorders, Peripheral neuropathy, Anti-ganglioside antibody, Undifferentiated connective tissue disease

## Abstract

**Background:**

Neuromyelitis optica spectrum disorders (NMOSD) is a group of autoimmune-mediated disorders of the central nervous system primarily involving the optic nerve and spinal cord. There are limited reports of NMOSD associated with peripheral nerve damage.

**Case presentation:**

We report a 57-year-old female patient who met the diagnostic criteria for aquaporin 4 (AQP4)-IgG positive NMOSD with undifferentiated connective tissue disease and multiple peripheral neuropathy. In addition, the patient was positive for multiple anti-ganglioside antibodies (anti-GD1a IgG antibodies and anti-GD3 IgM antibodies) and anti-sulfatide IgG antibodies in serum and cerebrospinal fluid. After treatment with methylprednisolone, gamma globulin, plasma exchange, and rituximab, the patient’s status improved and was subsequently discharged from our hospital.

**Conclusions:**

The neurologist should be aware of the unusual association between NMOSD and immune-mediated peripheral neuropathy undifferentiated connective tissue disease and nerve damage mediated by multiple antibodies may have combined to cause peripheral nerve damage in this patient.

## Introduction

Neuromyelitis optica spectrum disorders (NMOSD) is a group of autoimmune-mediated disorders of the central nervous system (CNS) characterized by recurrent attacks of the optic nerve and spinal cord. NMOSD is more common in middle-aged females with mean first age of onset at approximately 40 years of age [1]. The prevalence of NMOSD in East Asia is about 3.5/100000 [2]. Currently, most studies believe that the pathogenesis of NMOSD is related to the Aquaporin 4 (AQP4) antibody, which is expressed in the foot process of astrocytes distributed along the blood–brain barrier and the fovea of the retina [3, 4]. The autoimmune reaction caused by the interaction between AQP4 and its antibodies leads to astrocytic damage, eventually causing clinical symptoms. Approximately 4% of NMOSD patients have a monophasic course, yet the majority of NMOSD patients experience recurrent episodes of optic nerve and spinal cord damage(up to 60%-98% recurrence rate).NMOSD causes severe visual impairment (about 60%) and disability (about 34%) in young adults [4, 5], making it a focus of attention of neurologists. Herein, we report one case of AQP4-positive NMOSD coexisting with undifferentiated connective tissue disease and peripheral neuropathy. In addition, the patient was positive for multiple anti-ganglioside antibodies and anti-sulfatide IgG antibodies.

## Case presentation

A 57-year-old female patient was admitted to our hospital due to “nausea and vomiting for more than 4 months, numbness of limbs for more than 3 days, and blurred vision for 1 day.” Four months before admission, the patient developed nausea, hiccups, and vomiting without obvious inducement, and experienced unintentional weight loss. Her gastrointestinal endoscopy results were unremarkable, and her symptoms healed spontaneously without treatment. One month before admission, the patient began to experience numbness, itching, and tingling on the top of the head, as well as walking instability. She gradually developed numbness in her left upper limb and the inferior surface of the left anterior superior iliac spine, which continued without relief. Twenty days before admission, the patient developed bilateral facial numbness, and she was treated with drugs, such as pregabalin and mecobalamin, yet her symptoms did not improve. Three days before admission, the patient developed numbness and weakness in all four extremities. Two days later, her weakness worsened, accompanied by an unsteady gait, blurred vision, and occasional diplopia. During the course of the disease, the patient had no other presentations, such as dizziness, dysphagia, dyspnea, or dysphoria, and she denied history of chronic diseases, such as diabetes and rheumatic immune diseases.

On admission, physical examination of the nervous system revealed the following abnormalities: slight decrease in calculation ability and recent memory loss; reduced binocular visual acuity, diplopia, and horizontal coarse nystagmus in both eyes; spasmodic hypertonia of lower limbs; reduced (grade 4) muscle strength of lower limbs and distal end of upper limbs; segmental attenuation-disappearance of bilateral acupuncture sensation (from left thyroid cartilage to subclavian fossa, left upper limb, left anterior superior iliac spine below; from right mandibular angle to sternum); abdominal reflexes disappeared, limb tendon reflexes were hyperactive (+ + +), and clonus was present in bilateral ankles; bilateral finger-nose test and heel-knee-tibia test were inaccurate, and Romberg test was positive; Rossolimo sign was positive on the right side ( +), Babinski sign and Chaddock sign were positive on both sides ( +), and skin scratch sign was positive.

**Fig. 1:**
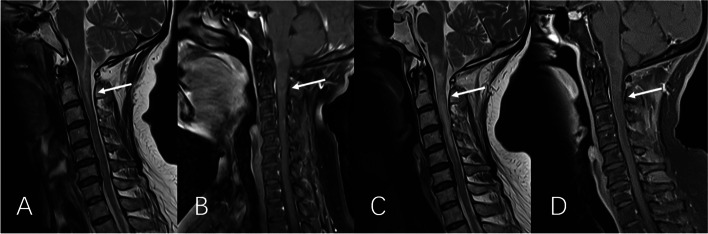
MRI findings of cervical cord. **A** and **B **Prior to treatment, MRI showed myelopathyfrom medulla oblongata to spinal cord C3; **C** and **D **MRI reexamination showed that the range of myelopathy was enlarged to spinal cord C5. White arrow indicates the lesion. A and C are T2-weighted images, while B and D are T1-weighted images

Auxiliary examinations showed positive antinuclear antibodies (ANAs): karyotype 1 (nucleolar type) with titer of 1:1000, karyotype 2 (cytoplasmic granular type) with titer of 1:100, anti-mitochondrial M2 antibodies were weakly positive, and anti-Ro-52 antibodies were positive. Lumbar puncture showed lower intracranial pressure (70mmH_2_O) and abnormal cerebrospinal fluid (CSF) results (nuclear cells: 92 × 10^6^/L, mononuclear cells: 89 × 10^6^/L, multinucleated cells: 3 × 10^6^/L; protein: 0.60 g/L, immunoglobulins G (IgG): 51.110 mg/L, IgM: 2.170 mg/L and IgA: 7.680 mg/L; positive for anti-sulfatide IgG antibodies and anti-AQP4 antibodies). In addition, she tested positive for anti-sulfatide IgG antibodies, anti-GD1a IgG antibodies, anti-GD3 IgM antibodies, and anti-AQP4 antibodies in her serum samples. More type III oligoclonal bands were seen in the CSF sample compared with the serum sample. Anti-myelin oligodendrocyte glycoprotein (anti-MOG) antibodies in serum and CSF samples were negative. Other examinations were almost normal. Three days after admission, neuroelectrophysiological examination demonstrated longer latency of the compound muscle action potential (CMAP), reduced occurrence rate of the F wave with prolonged latency, decreased sensory conduction velocity (SCV), and reduced sensory nerve action potential (SNAP) amplitude in the right wrist distribution of the median nerve; reduced CMAP and SNAP amplitudes, reduced occurrence rate of F wave with prolonged latency, decreased SCV and motor nerve conduction velocity (MCV) in the right ulnar nerve, presence of conduction block in the right common peroneal nerve and the tibial nerve, and decreased SCV in the right superficial peroneal nerve and the sural nerve (Table 1). One day later, spinal magnetic resonance imaging (MRI) revealed slight swelling with abnormal signals from medulla oblongata to spinal cord C3 (Fig. 1A and B), which were worsened at reexamination 33 days later (Fig. 1C and D). Results of other tests such as orbital MRI, parotid gland ultrasound, Schirmer test, and tear film breaking time were all normal or negative.

**Table 1:** Neuroelectrophysiological examination of right extremities before treatment

**Nerve**	**Stimulation** **site**	**Recording** **site**	**Amplitude**	**Latency (ms)**	**Conduction** **velocity(m/s)**	**F wave** **(ms)**
**Motor**						
Median	Wrist	APB	8.35	**4.45**	-	**32.4**
	Elbow	APB	7.71	8.70	54.10	-
	Axilla	APB	**5.51**	11.1	60.40	-
Ulnar	Wrist	ADQ	**2.92**	3.30	-	**37.3**
	Below elbow	ADQ	**1.80**	7.25	**48.1**	-
	Above elbow	ADQ	**1.96**	10.10	**34.5**	-
	Axilla	ADQ	**1.38**	12.00	**52.6**	-
Peroneal	Ankle	EDB	7.39	3.45	-	46.6
	Below fibular	EDB	6.37	10.30	44.90	-
	Above fibular	EDB	**3.38**	12.40	51.50	-
Tibial	Ankle	AHB	8.43	3.60	-	54
	Popliteal fossa	AHB	**3.35**	11.9	49.4	-
**Sensory**						
Median	Wrist	Second finger	**2.84**	3.67	**43.60**	-
Ulnar	Wrist	Fifth finger	**1.74**	3.47	**43.30**	-
Sural	Calf	Lateral malleolus	5.70	3.37	**37.10**	-
Peroneal	Lateral leg	Foot	**3.55**	3.57	**36.40**	-

Amplitudes are measured in millivolt (mV, motor) and in microvolt (µV, sensory). *APB* Abductor pollicis brevis, *ADQ* Abductor digitiquinti, *EDB* Extensor digitorum brevis, *AHB* Adductor halluces brevis

Based on all examination results, the patient was diagnosed as NMOSD coexisting with undifferentiated connective tissue disease and peripheral neuropathy. Her limb numbness and blurred vision were slightly improved after treatment with intravenous methylprednisolone (first dose of 1000 mg/d and then gradually reduced) and gamma globulin shock therapy (for 5 consecutive days). During treatment, the patient presented with type II respiratory failure, and invasive ventilation was used to assist ventilation. Sixteen days after admission, reexamination showed that AQP4 titers of serum and CSF were unchanged as compared with admission (titer of 1:32). Hormonotherapy was continued and a high dose of plasma exchange(2000 ml) was performed every other day for 5 times. The patient's respiratory condition was gradually improved and invasive ventilator assisted ventilation was no longer required. Oxygen inhalation in the tracheal intubation tube could maintain normal oxygen saturation, and the tracheal intubation was discontinued 3 days later. After two doses of intravenous rituximab (first dose: 100 mg and second dose: 500 mg), the patient discharged from our hospital with a better health condition. His neuroelectrophysiological examination results before discharge indicated that the damaged peripheral nerve had basically recovered (Table 2). At discharge, her limb numbness and blurred vision were improved, and she could walk with assistance (muscle strength of limbs was grade 5). She complained of spastic pain in her limbs, and slight horizontal nystagmus was still visible in both eyes during physical examination.-

**Table 2:** Re-examined neuroelectrophysiological examination of right extremities after treatment

Nerve	Stimulation site	Recording site	Amplitude	Latency (ms)	Conduction velocity (m/s)	F wave (ms)
**Motor**						
Median	Wrist	APB	20.82	3.46	-	27.25
	Elbow	APB	14.97	6.20	56.42	-
	Axilla	APB	10.95	8.48	62.44	-
Ulnar	Wrist	ADQ	18.61	2.24	-	27.51
	Below elbow	ADQ	14.87	4.34	64.18	-
	Above elbow	ADQ	14.68	6.24	55.26	-
	Axilla	ADQ	13.22	8.00	52.84	-
Peroneal	Ankle	EDB	17.68	3.62	-	44.75
	Below fibular	EDB	16.45	8.58	50.40	-
	Above fibular	EDB	17.39	10.34	46.59	-
Tibial	Ankle	AHB	15.84	3.04	-	47.14
	Popliteal fossa	AHB	14.70	10.70	49.33	-
**Sensory**						
Median	Wrist	Second finger	2.16	15.84	64.44	-
Ulnar	Wrist	Fifth finger	1.86	10.09	65.89	-
Sural	Calf	Lateral malleolus	2.70	14.96	50.21	-
Peroneal	Lateral leg	Foot	2.71	11.63	52.92	-

Amplitudes are measured in millivolt (mV, motor) and in microvolt (µV, sensory). *APB* Abductor pollicis brevis, *ADQ* Abductor digiti quinti, *EDB* Extensor digitorum brevis, *AHB* Adductor halluces brevis

## Discussion

In this case, the patient was positive for antinuclear antibodies, anti-mitochondrial M2 antibodies, and anti-Ro-52 antibodies, indicating the existence of autoimmune disease; however, her results of other tests such as parotid gland ultrasound and Schirmer test were all negative, and there were no signs of multi-tissue and organ damages. Therefore, she was diagnosed as undifferentiated connective tissue disease [4]. Historically, the complications of connective tissue disease were generally considered to be a direct cause of optic nerve and spinal cord transverse damage. It was reported that 25–44% of NMOSD patients had positive antinuclear antibodies [3], and NMOSD may coexisted with a variety of connective tissue diseases, such as systemic lupus erythematosus and Sjogren's Syndrome [6]. Combining the core clinical characteristics and coexisting diseases such assystemic lupus erythematosus and Sjogren's Syndrome will strengthen the diagnosis of NMOSD [7]. Recent studies found that AQP4-IgG can present alone or in conjunction with other autoimmune disease antibodies in the patient’s body, indicating the coexistence relationship between NMSOD and autoimmune diseases. In addition, AQP4-IgG positive NMOSD patients have an increased susceptibility to multiple autoimmune diseases [8]. The manifestations of this case may be connected with her undifferentiated connective tissue disease. We deduced that her immunologic derangementcondition caused by undifferentiated connective tissue disease may aggravate the adverse impacts of NMOSD on both CNS and peripheral nervous system (PNS).

NMOSD with peripheral neuropathy, although rare, has been reported as early as 1991 [9]. Currently, the pathogenesis of peripheral neuropathy in NMOSD remains unclear. Seungyeon Kim et al. have proposed two different viewpoints [10]: First, AQP4 exists in the nerve root, atransitional zone of CNS and PNS [11], which may be used to explain the nerve root damage found in NMOSD patients. Second, there may be some other unknown antibodies to axoglial antigen that also play a role in PNS damage. Kato J et al. speculated that, in serum AQP4-IgG- positive NMOSD patient, PNS damage may be induced by AQP4-antibody-mediated astrocyte dysfunction or by peripheral AQP4-antigen-associated complement activation [12]. Feyiss et al.believed that blood–brain barrier disruption caused by locally reduced blood flow, hypoxia, and serious inflammation could be contributing to PNS damage in NMOSD cases [13]. It has also been reported that peripheral neuropathy was mediated by immune mechanisms caused by other antibodies, humoral factors or infected viruses [14, 15]. This viewpoint was in line with the findings of a case report of Charcot-Marie-Tooth Disease Type 1Acomplicated by NMOSD [16]. In that case, authors speculated that the patient’s PNS damage may be caused by an immune cross-reaction induced by over-expression of peripheral myelin protein 22 in the peripheral nerve. Gangliosides are a large family of sialic acid-containing glycosphingolipids that act as receptors for axon-glia interactions required for cytoskeletal structure stabilization and axonal regeneration. Therefore, binding of anti-ganglioside antibodies may lead to delay of nerve repair [17]. Previous evidence has suggested thatanti-GD1a antibodies and anti-GD3antibodies are closely associated with the occurrence and development of peripheral nerve myelin sheath injury in Guillain–Barre syndrome.Anti-GD1a antibodies also participate in the development of Miller-Fisher syndrome [18]. Sulfatide is the main component of myelin in CNS and PNS [19]. Axonal demyelination caused by anti-sulfatide antibodies often involves sensory neuropathy and is more common in autoimmune peripheral neuropathy, which often manifests as symmetrical distal limb numbness, weakness, and sensory ataxia [20]. Currently, only one case of NMOSD with peripheral neuropathy has reported an elevated GD1b level [10]. From this perspective, our case is very unique because she was positive for anti-GD3 antibodies, anti-GD1a IgG, antibodies and anti-sulfatide antibodies.

Intravenous methylprednisolone (IVMP) is used as first-line treatment of acute attacks of NMOSD, Plasma exchange (PLEX) or immunoadsorption are recommended within 5 days from NMOSD relapse onset, when response to IVMP is poor or absent. PLEX can also be administered as first-line therapy or simultaneously with IVMP in severe cases [21, 22]. Intravenous immunoglobulins (IVIg) is generally not the preferred treatment for NMOSD unless patients with contraindications to IVMP and apheresis therapies [22]. Immunosuppressive treatments to prevent relapse in NMOSD is especially important, which is recommended to start treatment as early as possible after diagnosis and to adhere to long-term treatment [21, 22]. In this case, the patient’s clinical symptoms improved after treatment with methylprednisolone, gamma globulin, plasma exchange, and rituximab. Therefore, we suggest that, For the NMOSD patients with peripheral neuropathy, IVMP combined with IVIg or PLEX may be a better treatment option.

## Conclusions

This is the first case report of NMOSD with peripheral neuropathy coexisting with undifferentiated connective tissue disease, which had also tested positive for multiple anti-ganglioside antibodies and anti-sulfatide IgG antibodies. An immunologic derangement condition caused by undifferentiated connective tissue disease and multiple antibody-mediated neurological insults may have jointly contributed to her peripheral damage.

## Data Availability

Data are available from the Yangchun Li upon reasonable request and with permission of Guizhou Provincial People’s Hospital.
